# Qualitative radiographic characteristics of MRONJ-affected bone in oral and parenteral drug administration: comparison of panoramic radiography and cone-beam CT

**DOI:** 10.1186/s12903-025-06463-8

**Published:** 2025-07-02

**Authors:** Elif Aslan, Erinc Onem, Ali Mert, B. Guniz Baksi

**Affiliations:** 1 Department of Oral and Maxillofacial Radiology, Ege University School of Dentistry, Izmir, Turkey; 2 Department of Engineering Sciences, Izmir Katip Celebi University School of Engineering and Architecture, Izmir, Turkey

**Keywords:** Medication-related osteonecrosis of the jaw, Cone-beam computed tomography, Panoramic radiography

## Abstract

**Background:**

Our aim was to compare the capacity of panoramic radiography (PR) and cone-beam computed tomography (CBCT) first for the detection of bone affected by medication-related osteonecrosis of the jaw (MRONJ) using qualitative radiographic parameters; and second for the evaluation of the relationship between drug administration route and associated bone changes.

**Methods:**

PR and CBCT images of 24 MRONJ patients using oral or intravenous (IV) bisphosphonates were scored for the detection of osteolysis, osteosclerosis, lamina dura thickening, periodontal ligament widening, persistent extraction socket, sequestration, periosteal new bone formation, narrowing of the inferior alveolar canal, pathologic fracture, and cortical bone erosion. Fisher’s Exact and independent samples t-tests were used for comparisons (*p* = 0.05).

**Results:**

The most frequent parameters observed in PR of all patients were osteosclerosis, osteolysis and cortical bone erosion, respectively. The equivalent rank in CBCT was cortical bone erosion, osteosclerosis and osteolysis. CBCT showed better diagnostic performance for the detection of periosteal new bone formation and cortical bone erosion in both oral and IV bisphosphonate groups (*p* < 0.05). No difference was obtained between the diagnostic performances of two imaging methods for the discrimination of the remaining parameters (*p* > 0.05). A higher number of radiographic markers were detected in both PR and CBCT images of patients using IV bisphosphonates (*p* > 0.05).

**Conclusion:**

In this study, the diagnostic performance of PR was comparable to CBCT for the detection of most of the qualitative radiographic markers of MRONJ-affected bone in patients using oral and IV bisphosphonates. However, CBCT may be preferred for the diagnosis of periosteal new bone formation, cortical bone erosion, and pathologic fracture.

## Introduction

Medication-related osteonecrosis of the jaws (MRONJ) is an adverse effect of anti-resorptive and anti-angiogenic drugs characterized by the progressive destruction of alveolar bones [1]. The severity of MRONJ and the amount of bone destruction affect treatment planning and accordingly, the prognosis and success of the treatment [2]. However, the diversity of clinical findings of MRONJ necessitates the use of imaging for conclusive diagnosis and for more detailed evaluation and detection of alveolar bone changes [3]. Therefore, the use of panoramic radiography (PR) and cone-beam computed tomography (CBCT) has been recommended for the detection of bone changes due to MRONJ [4].

The American Association of Oral and Maxillofacial Surgeons (AAOMS) updated their position paper in 2022 and included a list of radiographic features linked to MRONJ [2]. These include osteolysis, osteosclerosis, thickening of the lamina dura (LD), widening of the periodontal ligament (PDL), persistent extraction socket, sequestration, periosteal new bone formation, narrowing of the inferior alveolar canal, and pathologic fracture In addition to these parameters, recent studies have suggested the use of mandibular cortical erosion in order to reveal the development of the disease, and this parameter has also been considered decisive for the diagnosis of MRONJ [5, 6].

Numerous studies have well-proven the superiority of three-dimensional (3D) imaging over PR in detecting structural bone changes [7–11]. Therefore, the experts particularly recommend the use of CBCT as cross-sectional images as cross-sectional images can detect micro-architectural bone changes before 2D images reveal them [12]. Despite the high number of studies comparing PR and CBCT for their diagnostic capacity in terms of delineation of most of the qualitative radiographic findings of MRONJ [9, 11, 13–15], the detection of mandibular cortical erosion has not been previously investigated. In fact, studies comparing PR and CBCT for the detection of MRONJ-affected bone using qualitative findings have included the evaluation of only a few radiographic parameters, but no study has combined the assessment of all defined qualitative parameters [9, 11, 13–15]. Furthermore, there has not been a thorough study using CBCT images to examine the relationship between the drugs’ administration route and the associated radiographic bone changes related to MRONJ.

Therefore, the aim(s) of this study were to compare the capacity of PR and CBCT images for (1) the detection of ten different qualitative radiographic parameters of MRONJ including mandibular cortical erosion, and (2) the evaluation of the relationship of the route of administration of the drugs and associated bone changes.

## Materials and methods

### Ethical approval

The present study was approved by the Institutional Ethics Committee (17.05.2022/22-5T/5) of the University and followed the principles of the Declaration of Helsinki.

### Selection of MRONJ patients

Medical history of patients referred to the Department of Oral and Maxillofacial Radiology between 2018 and 2020 was obtained from the patient database using the keywords bisphosphonate, cancer, osteoporosis, and osteonecrosis. Thousand patients containing at least one of these keywords in their data were selected and examined in terms of the type of medications. Patients using anti-resorptive and/or anti-angiogenic drugs but not clear about the drug type, and patients with a history of radiotherapy to the head and neck region and/or having metastases to the jawbones were excluded.

Among the remaining 278 patients, 24 (16 females, 8 males) with a mean age of 68.1 ± 11.2 who had been using anti-resorptive/anti-angiogenic drugs with histopathologically confirmed MRONJ in the mandible and had both PR and CBCT images in the archive were selected and included in the study. Based on their medical histories, eight of the 24 patients had a previous diagnosis of osteoporosis, while the remaining 16 were on bisphosphonates for breast (*n* = 7), prostate (*n* = 6) or lung cancer (*n* = 1), or multiple myeloma (*n* = 2). The type of intravenous bisphosphonate used for treatment was zoledronate in 45.8% (*n* = 11) of the patients, alendronate in 8.3% (*n* = 2) and ibandronate in 4.2% (*n* = 1). In 10 patients (41.7%), the type of oral bisphosphonate was not specified.

The MRONJ stage of the patients was established using the patient database, which included dental history, oral findings and radiographic images. MRONJ staging was done in agreement with the latest guidelines of the AAOMS [2]. Accordingly, nineteen out of 24 patients were classified as Stage 2, while the remaining five were Stage 3 MRONJ patients.

While CBCT volumes of either the right or left mandible were available for 16 patients, entire mandible volume was available for the remaining eight patients. Therefore, 14 hemi-mandibles of 10 MRONJ patients (7 females, 3 males; mean age: 69.6 ± 12.49) using oral bisphosphonate and 18 hemi-mandibles of 14 MRONJ patients (9 females, 5 males; mean age: 67.43 ± 11.02) receiving IV bisphosphonate were included. Consequently, the evaluations utilized CBCT images of a total of 32 hemi-mandibles from 24 MRONJ patients, along with their equivalent PR images.

### Image acquisition

PR images of patients had been previously obtained with Kodak 8000 digital PR device (Kodak Carestream Health Inc., Trophy, France) using 68 kV, 8 mA, and 13.9 s exposure parameters. Similarly, CBCT images had been previously obtained using the Kodak 9000 3D (Kodak Carestream Health, Trophy, France) device with exposure parameters of 70 kV, 10 mA, 10.8 s, and 76 μm (50 × 37 mm FOV) or 200 μm (85 × 37 mm FOV) isotropic voxel size.

### Image evaluation

The evaluation of the images focused on the mandibular posterior regions of both PR and CBCT images, aiming to eliminate the disadvantages of superimpositions in the anterior region of PR and the higher incidence of MRONJ in the posterior regions. PR and CBCT images of MRONJ patients were visually scored for the presence of ten qualitative radiographic parameters: (1) Osteolysis, (2) osteosclerosis, (3) LD thickening, (4) PDL widening, (5) persistent extraction socket, (6) sequestration, (7) periosteal new bone formation, (8) narrowing of the inferior alveolar canal, (9) pathologic fracture, and (10) mandibular cortical erosion.

Selected qualitative parameters was evaluated using sagittal and axial CBCT slices. Three oral and maxillofacial radiologists with three, seven, and eleven years of experience (EA, EO, BGB) used a binary scale to score the above-listed qualitative parameters in PR and CBCT images. In order to eliminate observer bias, PR and CBCT images were scored four weeks apart. The radiographic findings were scored as “0” when the finding was absent and “1” when the finding was present. For each imaging modality the parameters that were scored as “1” in consecutive evaluations were accepted as “present”.

A single observer (EA) scored both PR and CBCT images for the second time, four weeks after the initial evaluation, to assess intra-observer reliability.

**Fig. 1:**
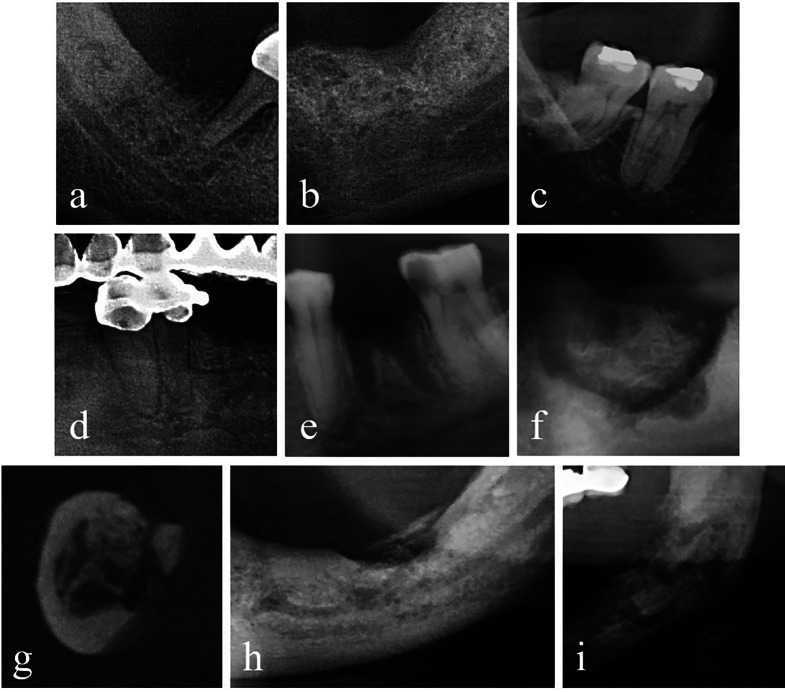
Representative PR and CBCT images demonstrating qualitative radiographic parameters. Cropped PR images showing (**a**) osteolysis, (**b**) osteosclerosis, (**c**) LD thickening, (**d**) PDL widening, (**e**) persistent extraction socket, (**f**) sequestration. (**g**) Cropped coronal CBCT image showing periosteal new bone formation. Cropped PR images demonstrating (**h**) narrowing of the inferior alveolar canal and (**i**) pathologic fracture

Osteolysis, osteosclerosis, LD thickening, PDL widening, persistent extraction socket, sequestration, periosteal new bone formation, narrowing of the inferior alveolar canal, and pathologic fracture (Fig. 1) were evaluated based on the definitions suggested by Ruggiero et al. [2], and Leite et al. [16].

Mandibular cortical bone erosion was evaluated according to the mandibular cortical index classification suggested by Klemetti et al. [17].

**Fig. 2:**
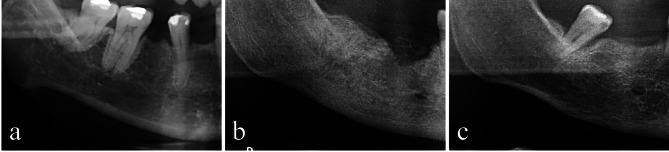
Cropped PR images showing different levels of mandibular cortical bone degeneration. (**a**) Normal cortex with a sharp and even endosteal margin (**b**) mild-to-moderate cortex erosion with semilunar defects in the endosteal margin and (**c**) severely porous cortical layer

- Cl: The endosteal margin of the cortex is even and sharp on both sides.
- C2: The endosteal margin shows semilunar defects (lacunar resorption) or endosteal cortical residues (one to three layers) on one or both sides.
- C3: The cortical layer is clearly porous and has heavy endosteal cortical residues (Fig. 2).

Accordingly, patients with endosteal cortical margins showing C2 or C3 types of defects were considered as having mandibular cortical bone erosion.

### Statistical analysis

Data analysis was performed using the IBM SPSS Statistics 20.0 (SPSS Inc., Chicago, IL, United States). The Fisher’s Exact test was used to evaluate the capacity of PR and CBCT for the delineation of all qualitative parameters. The comparison was done for all patients, oral and IV drug users separately (*p* = 0.05). The comparison of groups using oral or IV bisphosphonates with regard to the frequency of qualitative radiographic parameters for both PR and CBCT images was done using the independent samples t-test (*p* = 0.05).

The inter-observer reliability among three observers (EA, EO, BGB) as well as the intra-observer reliability were determined using the Cohen’s Kappa test (κ).

## Results

### Comparison of PR and CBCT images for the detection of qualitative radiographic parameters in all MRONJ patients

Most frequent qualitative parameters found in PR images were osteosclerosis (75%), osteolysis (69%), and cortical bone erosion (66%), respectively, while the equivalent ranking in CBCT images was cortical bone erosion (100%), osteosclerosis (78%), and osteolysis (69%) (Table 1).

CBCT images revealed higher diagnostic performance than PR images for the discrimination of periosteal new bone formation (*p* < 0.0001) and cortical bone erosion (*p* < 0.0001) (Table 1). However, there was no difference between the scores of the two imaging modalities in the diagnosis of osteolysis, osteosclerosis, LD thickening, PDL widening, persistent extraction socket, sequestration and narrowing of the inferior alveolar canal (*p* > 0.05) (Table 1).

**Table 1:** Scores (n) and percentages (%) of all qualitative radiographic parameters (QRPs) obtained using PR and CBCT images of all MRONJ patients using bisphosphonates

QRPs	PR *n* (%)	CBCT *n* (%)	*p* value
Osteolysis	22 (69)	22 (69)	1
Osteosclerosis	24 (75)	25 (78)	1
LD thickening	11 (34)	12 (38)	1
PDL widening	9 (28)	12 (38)	0.60
Persistent extraction socket	7 (22)	7 (22)	1
Sequestration	7 (22)	8 (25)	1
Periosteal new bone formation	0 (-)	14 (44)	0.000*
Mandibular cortical bone erosion	21 (66)	32 (100)	0.000*
Narrowing of the inferior alveolar canal	13 (41)	17 (53)	0.45
Pathologic fracture	0 (-)	3 (9)	0.24

LD: Lamina dura, PDL: Periodontal ligament, PR: Panoramic radiography, CBCT: Cone-beam computed tomography. *p < 0.05

**Fig. 3:**
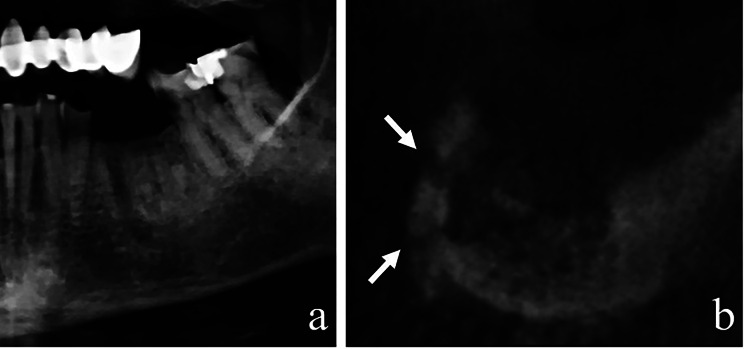
(**a**) A cropped PR image showing osteonecrosis in the mandibular posterior region. (**b**) A cropped coronal CBCT image showing a pathologic fracture in the buccal cortical wall of the mandible (white arrows)

Pathologic fractures were observed only in three patients and all of them were identified in CBCT images, while none could be distinguished with PR (Fig. 3). However, due to the low number of patients showing pathologic fractures, no significant difference was obtained between the two imaging methods for the diagnosis of pathologic fracture (*p* > 0.05).

### Comparison of PR and CBCT images for the detection of qualitative radiographic parameters in MRONJ patients using oral bisphosphonates

The most common qualitative findings in PR images of MRONJ patients taking oral bisphosphonates were osteolysis (71%), osteosclerosis (64%), and cortical bone erosion (57%). The equivalent ranking in CBCT images was cortical bone erosion (100%), osteosclerosis (79%), and osteolysis (71%), respectively (Table 2).

CBCT demonstrated higher diagnostic performance in distinguishing mandibular cortical bone erosion in MRONJ patients using oral bisphosphonates (*p* = 0.008) (Table 2). The scores of the remaining 7 qualitative parameters showed no difference between PR and CBCT images (*p* > 0.05).

**Table 2:** PR and CBCT scores (n) and percentages (%) of qualitative radiographic parameters (QRPs) for MRONJ patients using oral bisphosphonates

QRPs	PR *n* (%)	CBCT *n* (%)	*p* value
Osteolysis	10 (71)	10 (71)	1
Osteosclerosis	9 (64)	11 (79)	0.42
LD thickening	7 (50)	8 (57)	0.72
PDL widening	5 (36)	7 (50)	0.46
Persistent extraction socket	2 (14)	4 (29)	0.38
Sequestration	3 (21)	3 (21)	1
Periosteal new bone formation	0 (-)	4 (29)	0.15
Mandibular cortical bone erosion	8 (57)	14 (100)	0.008*
Narrowing of the inferior alveolar canal	5 (36)	6 (43)	0.71
Pathologic fracture	0 (-)	1 (7)	0.34

LD: Lamina dura, PDL: Periodontal ligament, PR: Panoramic radiography, CBCT: Cone-beam computed tomography. *p < 0.05

### Comparison of PR and CBCT images for the detection of qualitative radiographic parameters in MRONJ patients using IV bisphosphonates

The most common qualitative finding in PR images of MRONJ patients receiving IV bisphosphonates was osteosclerosis (83%), followed by cortical bone erosion (72%) and osteolysis (67%). The comparable CBCT rating was cortical bone erosion (100%), osteosclerosis (78%), and osteolysis (67%) (Table 3).

The diagnostic performance obtained using CBCT images was superior to PR for the detection of periosteal new bone formation (*p* < 0.0001) and mandibular cortical bone erosion (*p* = 0.02) in MRONJ patients using IV bisphosphonates. No difference was obtained between the diagnostic performance of PR and CBCT images regarding the detection of the remaining parameters (*p* > 0.05).

**Table 3:** PR and CBCT scores (n) and percentages (%) of qualitative radiographic parameters (QRPs) for MRONJ patients using IV bisphosphonates

QRPs	PR *n* (%)	CBCT *n* (%)	*p* value
Osteolysis	12 (67)	12 (67)	1
Osteosclerosis	15 (83)	14 (78)	0.68
LD thickening	4 (22)	4 (22)	1
PDL widening	4 (22)	5 (28)	0.71
Persistent extraction socket	3 (17)	1 (6)	0.30
Sequestration	4 (22)	5 (28)	0.71
Periosteal new bone formation	0 (-)	10 (56)	0.000*
Mandibular cortical bone erosion	13 (72)	18 (100)	0.02*
Narrowing of the inferior alveolar canal	8 (44)	11 (61)	0.33
Pathologic fracture	0 (-)	2 (11)	0.16

LD: Lamina dura, PDL: Periodontal ligament, PR: Panoramic radiography, CBCT: Cone-beam computed tomography. *p < 0.05

### Comparison of MRONJ patients with regard to the route of administration of bisphosphonates

Osteolysis, osteosclerosis, persistent extraction socket, sequestration, mandibular cortical bone erosion and narrowing of the inferior alveolar canal were more prevalent in PR images of MRONJ patients using IV bisphosphonates. However, LD thickening and PDL widening were more frequently detected in PR images of patients using oral bisphosphonates. However, no significant difference was found for any of the evaluated qualitative parameters (*p* > 0.05).

CBCT images of MRONJ patients using IV bisphosphonates demonstrated higher scores for osteolysis, osteosclerosis, sequestration, periosteal new bone formation, mandibular cortical bone erosion, narrowing of the inferior alveolar canal and pathologic fracture. However, LD thickening, PDL widening, and persistent extraction socket scores were more prevalent in CBCT images of patients using oral bisphosphonates. No difference was obtained among the tested qualitative parameters (*p* > 0.05) (Table 4).

**Table 4:** Scores (n) and percentages (%) of qualitative radiographic parameters (QRPs) of MRONJ patients using IV and oral bisphosphonates for PR and CBCT images

	PR			CBCT			
QRPs	Oral *n* (%)	IV *n* (%)	*p* value		Oral *n* (%)	IV *n* (%)	*p* value
Osteolysis	10 (71)	12 (67)	0.78		10 (71)	12 (67)	0.78
Osteosclerosis	9 (64)	15 (83)	0.25		11 (79)	14 (78)	0.96
LD thickening	7 (50)	4 (22)	0.12		8 (57)	4 (22)	0.06
PDL widening	5 (36)	4 (22)	0.42		7 (50)	5 (28)	0.21
Persistent extraction socket	2 (14)	3 (17)	0.44		4 (29)	1 (6)	0.42
Sequestration	3 (21)	4 (22)	0.96		3 (21)	5 (28)	0.69
Periosteal new bone formation	0 (-)	0 (-)	0.34		4 (29)	10 (56)	0.14
Mandibular cortical bone erosion	8 (57)	13 (72)	0.39		14 (100)	18 (100)	-
Narrowing of the inferior alveolar canal	5 (36)	8 (44)	0.63		6 (43)	11 (61)	0.32
Pathologic fracture	0 (-)	0 (-)	-		1 (7)	2 (11)	0.71

LD: Lamina dura, PDL: Periodontal ligament, PR: Panoramic radiography, CBCT: Cone-beam computed tomography, IV: Intravenous

### Inter- and intra-observer reliability

Inter-observer and intra-observer kappa coefficients obtained for PR and CBCT images ranged between 0.81 and 1.00 for almost all qualitative parameters (Table 5). Since cortical bone degeneration was detected in all CBCT images and pathologic fracture could not be detected in any of the PR images, Kappa test could not be performed for these parameters.

**Table 5:** Inter- (κ_a_) and intra-observer (κ_b_) kappa coefficients of PR and CBCT images for all qualitative radiographic parameters (QRPs)

QRPs	Kappa_a_ (PR)	Kappa_a_ (CBCT)	Kappa_b_ (PR)	Kappa_b_ (CBCT)
Osteolysis	0.86	0.93	0.92	0.94
Osteosclerosis	0.91	1.00	0.92	1.00
LD thickening	1.00	1.00	0.96	1.00
PDL widening	1.00	1.00	0.94	1.00
Persistent extraction socket	1.00	1.00	1.00	1.00
Sequestration	0.91	0.86	0.93	0.87
Periosteal new bone formation	1.00	0.94	1.00	0.95
Mandibular cortical bone erosion	0.93	NA	0.95	NA
Narrowing of the inferior alveolar canal	0.87	1.00	0.91	0.97
Pathologic fracture	NA	0.84	NA	1.00

LD: Lamina dura, PDL: Periodontal ligament, PR: Panoramic radiography, CBCT: Cone-beam computed tomography, NA: Not applicable

## Discussion

The diverse and misleading nature of clinical findings of MRONJ has made radiographic imaging methods the primary choice for the detection of alveolar bone changes caused by this pathology. Researchers have asserted that quantitative bone parameters offer a more comprehensive and detailed understanding of structural bone alterations [18, 19]. However, studies have also proven that image noise, particularly in high-quality CBCT images with voxel resolution less than 0.1 mm, tends to affect the measurements of quantitative parameters [20–22]. Furthermore, the daily clinical routine acknowledges the time-consuming nature of quantitative parameter assessment, as each measurement necessitates the use of separate image analysis software, a series of image processing steps, and the selection of the most discriminatory anatomical region relevant to the pathology [23]. This long and difficult process necessitates the use of qualitative markers of MRONJ, as well as the selection of the best imaging method delineating the details of each marker essential for more practical detection of the pathology during clinical rush. Since there is no study comparing the diagnostic performances of PR and CBCT including the detection of all of the qualitative parameters of MRONJ, this is the first study evaluating all of the identified parameters as well as the mandibular cortical erosion. Furthermore, this study is novel as it evaluates the diagnostic capacity of PR and CBCT by examining the relationship between the drug’s administration route and associated bone changes.

According to our results, osteosclerosis and osteolysis were the most frequent qualitative parameters found in PR and the second and third most common in CBCT images. Further evaluation of the scores by grouping oral and IV bisphosphonate users separately did not reveal any difference other than the insignificant ranking in terms of the most frequently observed parameters for both PR and CBCT. Findings of the present study regarding the most common qualitative radiographic parameters were consistent with the results of numerous previous studies reporting that osteosclerosis and osteolysis are the most frequent qualitative radiographic findings of both early and advanced-stage MRONJ patients [9–11, 24–29]. Unlike previous studies that reported LD thickening as a common qualitative radiographic finding of MRONJ [26, 30, 31], the present study detected LD thickening in less than half of both PR and CBCT images. The dentition of the patients could be the reason for the lower detection rate of LD thickening. Since twenty of the 24 MRONJ patients included in this study had 5 to 14 missing teeth and the remaining four were completely edentulous, the number of teeth examined for LD evaluation was relatively small. Accordingly, the results of our study may be misleading in terms of LD thickening and MRONJ relationship. However, no study has yet evaluated the most common qualitative parameters based on the dentition status of patients. Therefore, further studies are needed to reveal the impact of dentition on the frequency of various qualitative radiographic findings of MRONJ.

The results of the present study revealed that mandibular cortical erosion was the most frequent qualitative parameter in CBCT, with a detection rate of 100%, regardless of the route of bisphosphonate administration. To our knowledge, there are two studies evaluating this parameter for the diagnosis of MRONJ using a reliable index [5, 6]. Although our finding was in agreement with the result of Kubo et al. [5], Ozcan et al. [6] reported a notably lower rate (40%) for mandibular cortical erosion in MRONJ patients using CBCT images with a voxel size of 300 μm. One of the primary reasons for the high rate of erosion found in the present study may be associated with the severity of bone lesions due to the advanced stage of the MRONJ patients. Another reason for the high rate of cortical erosion found in our study may be the voxel size, and accordingly, the high resolution of CBCT images (76 μm). Inclusion of high-resolution images in the evaluation of qualitative parameters resulted in convenient and accurate detection of degeneration in the endosteal margin of mandibular cortical bone. Given that CBCT demonstrated a comprehensive and superior diagnostic performance compared to PR, we particularly recommend its use for detecting cortical erosion and diagnosing MRONJ-affected bone in patients taking both oral and IV bisphosphonates.

CBCT had a better diagnostic performance than PR in the discrimination of periosteal new bone formation and pathologic fractures in MRONJ-affected bone. Moreover, all three pathologic fractures were readily demonstrated with CBCT images, while none can be established with PR. Similar to the results of previous reports, the current study revealed that PR failed to correctly detect both the periosteal new bone formation and pathologic fractures in MRONJ patients receiving oral and IV bisphosphonates [10, 11, 14]. This is evident given that periosteal bone formation typically occurs on the buccal or lingual surfaces of cortical bone, and PR is unable to show changes in the bucco-lingual direction. Additionally, CBCT provides high-contrast cross-sectional images with high isotropic spatial resolution [32].

Numerous studies have demonstrated the superiority of CBCT over PR in identifying osteosclerosis, osteolysis, and sequester formation in MRONJ patients [9, 11, 14]. Hence, the use of CBCT was recommended for the detection of qualitative radiographic parameters of MRONJ. However, overall evaluation of the current results including the assessment of all identified parameters revealed that while CBCT was superior in detecting cortical bone erosion, periosteal new bone formation and pathologic fractures, PR was equally sufficient for the discrimination of the most prominent qualitative markers of MRONJ-affected bone (osteolysis, osteosclerosis, LD thickening, PDL widening, persistent extraction socket, sequestration and narrowing of the inferior alveolar canal). Therefore, we can conclude that PR is a reliable tool for evaluating the aforementioned qualitative parameters of MRONJ-affected bone. This recommendation is particularly relevant considering that PR is more widely used in dental clinics with its relatively low radiation dose and cost compared to CBCT. However, it is crucial to note that if there is any uncertainty regarding the extent of the qualitative findings, it would be prudent to supplement the diagnosis with a CBCT to ensure accurate diagnosis.

The severity of MRONJ and therefore the extent and frequency of qualitative findings may vary depending on the route of administration of bisphosphonate drugs [1, 33]. Since IV bisphosphonates suppress osteoclastic resorption more effectively than oral bisphosphonates, patients using IV bisphosphonates tend to demonstrate more advanced osteonecrosis [1, 24, 34, 35]. Therefore, qualitative radiographic signs may be more significant and readily detected in patients taking more potent IV medications with higher destructive effect on bones [24, 34]. In agreement with these hypothesis, the results of the current study revealed that PR and CBCT images of patients using IV bisphosphonates demonstrated a higher number of qualitative radiographic parameters. However, LD thickening, PDL widening and persistent extraction socket were also detectable in PR and CBCT images of patients receiving oral bisphosphonates. In fact, despite the variabilities in the distribution of qualitative radiographic markers, diagnostic performance of PR was found to be equally adequate as compared to CBCT for the detection of most of the qualitative radiographic markers in both oral and IV drug groups.

Many studies have addressed the role of 3D diagnostics in the detection of necrotic bone changes due to oral or IV drug-induced MRONJ. According to Fedele et al. [36], up to 25% of all patients go undiagnosed due to the lack of radiological diagnostics. In this context, the AAOMS has included radiological signs in the recent classification in the updated position paper (2022) [2]. In the long term, the goal must be to define pathognomonic radiographic signs that signify bone alterations for the practitioner [28, 37]. Accordingly, the first task is to establish the best radiographic method for the detection of these bone alterations. To date, however, a small number of studies, including few numbers of qualitative parameters, have examined the capacity of PR and CBCT in the detection of bone changes due to MRONJ. The current findings suggest that qualitative radiographic markers may be useful in the detection of MRONJ-affected bone with a high degree of reliability. Moreover, we demonstrated that although CBCT was superior, PR was equally sufficient for the discrimination of the most prominent qualitative markers of MRONJ-affected bone in patients using both oral and IV bisphosphonates.

In the future, imaging may play a major role in patient care during or after anti-resorptive therapy. Determining distinct radiographic parameters that reliably identify affected bone regions could enhance patient dental therapy and potentially transform the entire follow-up strategy. Baseline PR images before medication, as well as regular follow-up examinations, may give information about bone changes. Early diagnosis can considerably reduce bone loss and consequently the long-term prognosis, which in turn influences quality of life [38, 39]. However, it is important to consider several limitations of the present study before making any recommendations regarding the imaging modality for the diagnosis of MRONJ-affected bone This study evaluated a relatively small number of MRONJ patients due to the strict inclusion criteria. Furthermore, the majority of the images in the image archive belonged to patients who had already been diagnosed with stage 2 and stage 3 MRONJ lesions, but there were no early-stage patients present who had both PR and CBCT images. Moreover, due to the lack of details in the patient database, the dental history of the patients prior to the development of MRONJ was missing, and therefore the relationship between risk factors leading to the osteonecrosis and imaging findings could not be interpreted. Therefore, further studies including “Risk Group” or “Stage 0” MRONJ patients with long-term follow-ups are required to support our recommendations. Nevertheless, the findings of the present study may provide the basis for further studies comparing the diagnostic capacity of CBCT and PR using the same qualitative radiographic parameters.

In conclusion, the most frequent qualitative parameters observed both in PR and CBCT images of MRONJ patients, regardless of the route of bisphosphonate administration, were osteosclerosis, osteolysis, and mandibular cortical bone erosion. However, patients using IV bisphosphonates demonstrated a higher number of radiographic markers. The findings of this study confirmed that the diagnostic performance of PR is comparable to CBCT for the detection of osteolysis, osteosclerosis, LD thickening, PDL widening, persistent extraction socket, sequestration and narrowing of the inferior alveolar canal in MRONJ patients. However, CBCT demonstrated a better diagnostic performance than PR for the detection of mandibular cortical bone erosion, pathologic fracture and periosteal bone formation, and therefore, may be preferred for the radiographic diagnosis of these particular findings in MRONJ-affected bone.

## Data Availability

The datasets generated and/or analysed during the current study are available from the corresponding author on reasonable request.

## Abbreviations

3D: Three dimensional
AAOMS: American Association of Oral and Maxillofacial Surgeons
CBCT: Cone-beam computed tomography
IV: Intravenous
LD: Lamina dura
MRONJ: Medication-related osteonecrosis of the jaw
QRPs: Qualitative radiographic parameters
PDL: Periodontal ligament
PR: Panoramic radiography
